# Amyloid‐β activates NLRP3 inflammasomes by affecting microglial immunometabolism through the Syk‐AMPK pathway

**DOI:** 10.1111/acel.13623

**Published:** 2022-04-27

**Authors:** Eun Sun Jung, Kyujin Suh, Jihui Han, Heyyoung Kim, Hyung‐Sik Kang, Won‐Seok Choi, Inhee Mook‐Jung

**Affiliations:** ^1^ Department of Biochemistry and Biomedical Sciences College of Medicine Seoul National University Seoul Korea; ^2^ Neuroscience Research Institute College of Medicine Seoul National University Seoul Korea; ^3^ School of Biological Sciences and Technology College of Natural Sciences Chonnam National University Gwangju Korea; ^4^ SNU Dementia Research Center College of Medicine Seoul National University Seoul Korea

**Keywords:** Alzheimer's disease, inflammasome, microglia

## Abstract

Neuroinflammation is considered one of major factors in the pathogenesis of Alzheimer's disease (AD). In particular, inflammasome activation, including NLRP3 inflammasome in microglia, is regarded as fundamental for the pro‐inflammatory response of immune cells. However, the precise molecular mechanism through which the NLRP3 inflammasome is associated with AD pathologies remains unclear. Here, we show that amyloid‐β activates the NLRP3 inflammasome in microglia by activating Syk and inhibiting AMPK. Deactivated AMPK induces metabolic dysregulation, mitochondrial fragmentation, and reactive oxygen species formation, leading to the activation of the NLRP3 inflammasome. In addition, flufenamic acid (FA), a member of non‐steroidal anti‐inflammatory drugs, was found to effectively inhibit activation of the microglial NLRP3 inflammasome by regulating Syk and AMPK. Importantly, FA has marked therapeutic effects on major AD pathologies and memory function in vivo in microglia‐dependent way. All together, these findings demonstrate the molecular mechanism of microglial NLRP3 inflammasome activation by amyloid‐β, which acts as an important mediator of neuroinflammation. Also, we suggest that repurposing of FA for inhibiting microglial activation of the NLRP3 inflammasome is a potential treatment for AD.

## Graphical abstract

In microglia, amyloid‐β caused Syk‐AMPK‐dependent mitochondrial dysfunction, which preceded NLRP3 inflammasome activation. Our study identified flufenamic acid (FA), a member of non‐steroidal anti‐inflammatory drugs, effectively inhibits the activation of the microglial NLRP3 inflammasome by regulating Syk and AMPK.

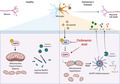

**Abbreviations**
Aβ: amyloid‐β
AD: Alzheimer’s disease
ASA: acetylsalicylic acid
ASC: apoptosis‐associated speck‐like protein containing a caspase recruitment domain
DAM: disease‐associated microglia
DAMP: damage‐associated molecular patterns
FA: flufenamic acid
FBS: fetal bovine serum
HBSS: Hank’s balanced salt solution
iBMDM: immortalized mouse bone marrow‐derived macrophages
IL: interleukin
MGnD: microglial neurodegenerative phenotype
NSAID: non‐steroidal anti‐inflammatory drugs
PAMP: pathogen‐associated molecular patterns
PDL: poly‐D‐Lysine
18S rRNA: 18S ribosomal RNA
ROS: reactive oxygen species
SyK: spleen tyrosine kinase

## 1 INTRODUCTION

Alzheimer's disease (AD), the most common type of neurodegenerative disease, is characterized by amyloid plaque, neurofibrillary tau tangles, and neuroinflammation, with the latter playing a crucial role in the progression of neuropathological hallmarks of AD (Heneka et al., 2015). As the most dominant immune cells in the brain, microglia are crucial in regulating neuroinflammation. Although amyloid‐β (Aβ) functions to primarily activate microglia in AD, its mechanism of activation is unclear. Activated microglia have been found to crowd around senile plaques in the brains of patients with AD (Ising & Heneka, 2018), suggesting that the microglial inflammatory response is associated with Aβ. In addition, various microglial receptors have been reported to interact with Aβ, including TREM2, a genetic risk factor for AD (Doens & Fernández, 2014). Disease‐specific subsets of microglia, such as disease‐associated microglia (DAM) (Deczkowska et al., 2018) and microglial neurodegenerative phenotype (MGnD) (Krasemann et al., 2017), have been identified in Aβ‐expressing animal models, indicating that Aβ is critical in stimulating immune responses in AD. Nevertheless, the exact mechanism by which Aβ induces microglial activation and affects the progression of AD remains unclear.

The present study investigated the mechanism by which Aβ activates microglia, focusing on the NLRP3 inflammasome of microglia. The NLRP3 inflammasome is involved in the activation and secretion of pro‐inflammatory cytokines, thereby acting as a key mediator of pro‐inflammatory responses in immune cells (Hanslik & Ulland, 2020). Activation of the NLRP3 inflammasome requires initiating and activating signals. Initiation signals, consisting of pathogen‐associated molecular patterns (PAMPs) or damage‐associated molecular patterns (DAMPs), induce the transcription of genes encoding pro‐interleukin (IL)‐1β and pro‐IL‐18. Subsequently, various activating signals, including ion flux, reactive oxygen species (ROS), and lysosomal damage, induce oligomerization of the sensor protein NLRP3, which recruits the adaptor protein, apoptosis‐associated speck‐like protein containing a caspase recruitment domain (ASC). Assembled ASC forms a multimeric complex, commonly called “speck,” which activates procaspase‐1 to cleave pro‐IL‐1β and pro‐IL‐18 proteins into their mature forms (Swanson et al., 2019). Both Aβ and tau have been reported to activate the NLRP3 inflammasome by inducing lysosomal damage (Halle et al., 2008; Stancu et al., 2019). This study shows that Aβ activates spleen tyrosine kinase (Syk), leading to the deactivation of AMPK and the activation of AKT. This signaling induces mitochondrial fragmentation and generation of ROS, thereby activating the NLRP3 inflammasome.

In addition, this signaling pathway is inhibited in microglia by flufenamic acid (FA), a type of non‐steroidal anti‐inflammatory drugs (NSAIDs), thereby regulating the Aβ‐induced activation of microglial inflammasome. NSAIDs have been widely used to treat many inflammatory diseases, but their therapeutic effect on AD remains unclear. This study found that FA, but not other classes of NSAIDs, ameliorates the activation of the microglial NLRP3 inflammasome, thereby effectively reducing neuropathological hallmarks of AD and restoring memory deficit.

## 2 RESULTS

### 2.1 Aβ42 activates the NLRP3 inflammasome via the Syk‐AMPK pathway in LPS‐primed microglia

**FIGURE 1:**
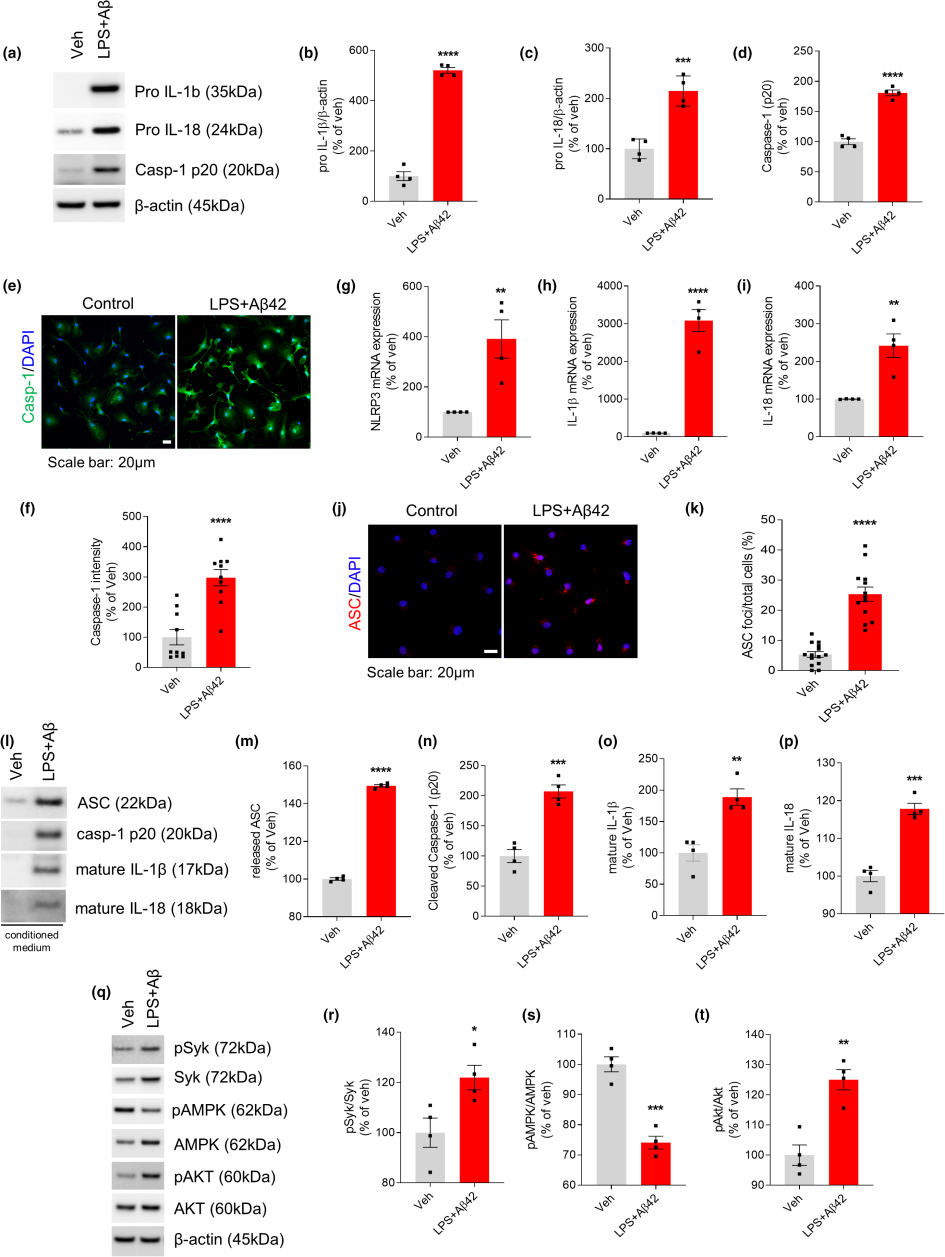
Aβ42 activates NLRP3 inflammasome in LPS‐primed microglia. Primary microglia were untreated (Veh) or primed with LPS (10 ng/ml, 3 h), followed by Aβ42 (4 μM) for 24 h. (a) Immunoblot showed that the protein expression levels of pro‐IL‐1β, pro‐IL‐18, and caspase‐1 (p20) were elevated after adding Aβ42 in LPS‐primed microglia. (b–d) Quantification of pro‐IL‐1β (b), pro‐IL‐18 (c) and caspase‐1 (d). (e) Representative immunofluorescence image of primary microglia with anti‐caspase‐1 (green). DAPI represents the nuclear signal (blue). Scale bar: 20 μm. (f) Quantification of immunofluorescence of caspase‐1. (g–i) The mRNA gene expression of NLRP3 (g), IL‐1β (h), and IL‐18 (i) was measured by real‐time PCR and expressed as relative fold change to vehicle group. (j) Immunofluorescence staining for ASC (red) speck. Scale bar: 20 μm. (k) Percentages of microglia containing ASC foci were quantified. (l) Immunoblot analysis of indicated proteins in conditioned media. Proteins from equal initial volumes were precipitated by TCA, and the levels of secreted proteins were quantified. (m–p) Quantification of secreted ASC (m), cleaved caspase‐1 (n), mature IL‐1β (o), and mature IL‐18 (p). (q) Immunoblot analysis of signaling proteins from cell lysates. (r–t) Quantitative analysis showed relative level of pSyk/Syk (r), pAMPK/AMPK (s), and pAkt/Akt (t). All data are shown as means ± SEM from four independent experiments and are analyzed by unpaired Student's *t* test. Confocal imaging data are from 5 randomly chosen fields per coverslip (e) or at least 200 cells per group (j). *p < 0.05, **p < 0.01, ***p < 0.001, and ****p < 0.0001

To determine whether Aβ42 activates inflammasome, we first assessed levels of key inflammasome components in primary microglia. Aβ42 drastically increased the levels of pro‐IL‐1β and pro‐IL‐18 in LPS‐primed microglia (Figure 1a–c). Similarly, LPS/Aβ42 enhanced caspase‐1 expression (Figure 1e,f) and immunoblotting showed that Aβ42 increases the level of active caspase‐1 (p20) (Figure 1d). In addition, stimulation of LPS‐primed microglia with Aβ42 significantly increased the levels of NLRP3, IL‐1β, and IL‐18 mRNAs compared with untreated microglia (Figure 1g–i). Immunofluorescence analysis showed that exposure of LPS‐primed microglia to Aβ42 enhanced the formation of ASC specks (Figure 1j,k). In addition, Aβ42 stimulation of microglia enhanced the secretion of mature IL‐1β, mature IL‐18, active caspase‐1 (p20), and ASC (Figure 1l–p). Taken together, these findings indicate that Aβ42 activates the NLRP3 inflammasome in LPS‐primed microglia.

Phosphorylation of Syk has been found to activate NLRP3 inflammasomes in macrophages and dendritic cells (Kankkunen et al., 2010; Thwe et al., 2019). Syk‐mediated activation of the PI3K/TBK1/Akt axis has been reported to drive glycolysis‐dependent IL‐1β production in dendritic cells in response to fungal‐associated beta‐glucan ligands (Gross et al., 2009). LPS/Aβ42 treatment of microglia induced striking increases in total Syk and expression of activated pSyk^Y525/526^ (Figure 1q–t). Recent evidence suggests that metabolic processes are associated with inflammatory responses of microglia (Ganeshan & Chawla, 2014). AMPK is regarded as a master regulator of metabolism, and Akt has important roles in many cellular processes, including metabolism. We found that LPS/Aβ42 promotes AMPK inactivation concomitant with Akt activation (Figure 1s,t).

### 2.2 Inhibition of Syk attenuates Aβ42‐induced inflammasome activation in LPS‐primed microglia

**FIGURE 2:**
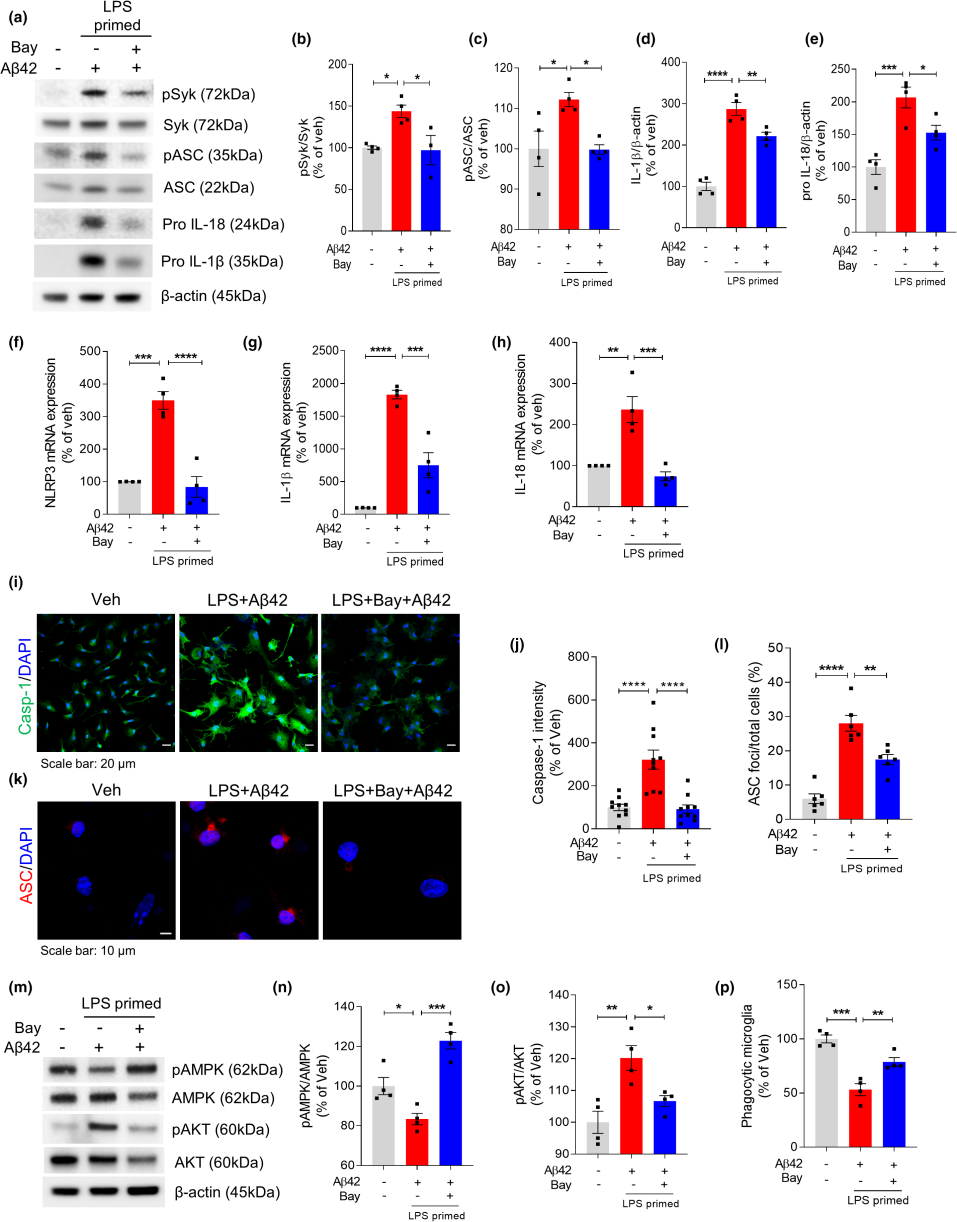
Inhibition of Syk protects Aβ42‐induced inflammasome activation in LPS‐primed microglia. LPS‐primed microglia were pretreated with Syk inhibitor Bay 61‐3606 for 1 h followed by treatment with Aβ42 for 24 h. (a–e) Representative image (a) and quantification (b–e) of Western blot for the pSyk, Syk, pASC, ASC, pro‐IL‐1β, IL‐18, and β‐actin. (f–h) mRNA expressions of NLRP3 (f), IL‐1β (g), and IL‐18 (h) were analyzed using real‐time PCR. 18S rRNA was used for normalization. (i) Representative immunofluorescence image of primary microglia with anti‐caspase‐1 (green). DAPI represents the nuclear signal (blue). Scale bar: 20 μm. (j) Quantification of immunofluorescence of caspase‐1. (k) Immunofluorescence staining for ASC (red) speck. Scale bar: 10 μm. (l) Percentages of microglia containing ASC foci. (m) Immunoblot analysis of indicated proteins in cell lysates. (n–o) Quantification of pAMPK (n) and pAKT (o) normalized by total AMPK and AKT, respectively. (p) Relative phagocytic activity of microglia measured by bead uptake assay. All data are presented as mean ± SEM from at least four independent experiments and analyzed by one‐way ANOVA and Tukey's multiple comparisons test. *p < 0.05, **p < 0.01, ***p < 0.001, and ****p < 0.0001

Because Aβ42‐induced NLRP3 inflammasome activation was associated with elevated levels of pSyk in LPS‐primed microglia, we evaluated the effect of the Syk inhibitor, BAY 61‐3606, on inflammasome activation. LPS‐primed microglia were pretreated with BAY 61‐3606 for 1 h and then stimulated with Aβ42. Although LPS/Aβ42‐stimulated microglia had increased levels of activated pSyk, pro‐IL‐1β, and pro‐IL‐18, pretreatment with BAY 61‐3606 strikingly inhibited the LPS/Aβ42‐induced increases in pSyk, pro‐IL‐1β, and pro‐IL‐18 levels. Syk activation was previously shown to induce ASC phosphorylation, enhancing ASC oligomerization (Lin et al., 2015). We confirmed that pASC is increased in microglia by LPS/Aβ42, an increase inhibited by treatment with Bay 61‐3606 (Figure 2a–e). Further, we found that Aβ42 stimulation markedly enhanced the production of NLRP3, IL‐1β, and IL‐18 mRNAs, but that these mRNA levels were significantly reduced by Syk inhibition in LPS‐primed microglia (Figure 2f–h). LPS/Aβ42‐induced inflammasome activation also increased caspase‐1 expression (Figure 2i,j) and ASC speck staining (Figure 2k,l), with both being significantly reduced by treatment with the Syk inhibitor BAY 61–3606. In testing the effect of Syk inhibitor on the activation of AMPK and Akt, we found that LPS/Aβ42‐induced reduction of pAMPK levels was restored by Syk inhibition. In addition, BAY 61–3606 treatment resulted in Akt dephosphorylation (Figure 2m–o). Evaluation of the effect of another Syk inhibitor, R406, showed that, similar to Bay 61–3606, R406 attenuated Aβ42‐induced inflammasome activation in LPS‐primed microglia (Figure S1). Furthermore, NLRP3 inflammasome activation by the Syk‐AMPK pathway affected phagocytic function of microglia. Bead uptake assays showed that LPS/Aβ42‐treated primary microglia showed lower phagocytic ability than vehicle‐treated microglia and that inhibition of Syk activation reversed this decrease in phagocytic activity (Figure 2p). Taken together, these findings show that Aβ activates Syk and inactivates AMPK in microglia.

### 2.3 Inhibition of Syk protects Aβ42‐induced mitochondrial fission in LPS‐primed microglia

**FIGURE 3:**
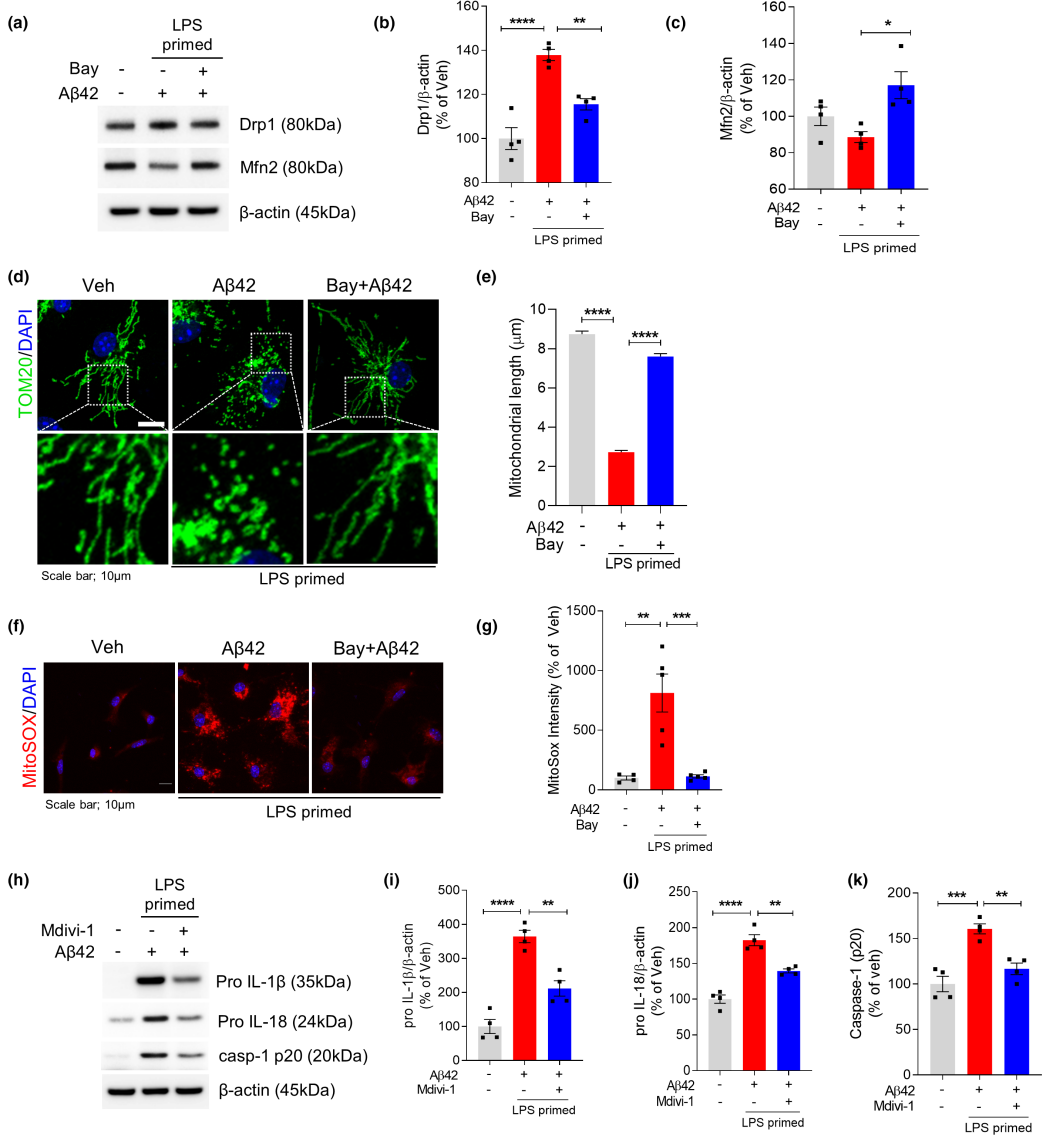
Inhibition of Syk protects Aβ42‐induced mitochondrial fission in LPS‐primed microglia. LPS‐primed microglia were pretreated with Syk inhibitor Bay 61‐3606 for 1 h followed by treatment with Aβ42 for 24 h. (a) Representative immunoblot of the protein levels of Drp1, Mfn2, and β‐actin in total cell lysate. (b and c) Densitometric quantifications showing the Drp1 (b) and Mfn2 (c). β‐Actin was used as a loading control. (d) Representative images of mitochondrial morphology, revealed by Tom20 staining. Mitochondria and nuclei were stained with Tom20 (red) and DAPI (blue), respectively. Scale bar: 10 μm. (e) Quantification of mitochondrial length. (f) Level of ROS in microglia stained with MitoSOX (red) and DAPI (blue) staining. Scale bar: 10 μm. (g) MitoSOX fluorescence intensity relative to veh was quantified. (h–k) LPS‐primed microglia were pretreated with Mdivi‐1, a selective mitochondria division inhibitor, for 1 h followed by treatment with Aβ42 for 24 h. pro‐IL‐1β (i), pro‐IL‐18 (j), caspase‐1 (p20) (k), and β‐actin levels were determined and quantified by Western blot. β‐Actin was used as a loading control. Data are shown as mean ± SEM from four independent experiments and are analyzed by one‐way ANOVA and Tukey's multiple comparisons test. *p < 0.05, **p < 0.01, ***p < 0.001, and ****p < 0.0001

Mitochondrial impairment is a strong activation signal for the NLRP3 inflammasome. Because AMPK is a master regulator of metabolism, we hypothesized that Syk‐AMPK signaling by Aβ induces mitochondrial damage, resulting in NLRP3 inflammasome activation. The fission/fusion balance is an important index of mitochondrial function, with imbalances between fission and fusion observed in various neurodegenerative diseases, including AD (Knott & Bossy‐Wetzel, 2008; Wang et al., 2009). We therefore investigated whether mitochondria are damaged by LPS/Aβ through Syk‐AMPK signaling. To investigate the mitochondrial fission/fusion balance in microglia, we measured the levels of Drp1 and Mfn2, proteins responsible for mitochondrial fission and fusion, respectively. LPS/Aβ42 increased the level of Drp1 and reduced the level of Mfn2, indicating excess mitochondrial fission. Interestingly, treatment with Syk inhibitor restored the levels of Drp1 and Mfn2 proteins (Figure 3a–c). Immunostaining of primary microglia with antibody to TOM20 also revealed the fission/fusion imbalance, as well as alterations in mitochondrial morphology. To determine the mitochondrial dynamics, we measured mitochondrial length. As shown in Figure 3b, LPS/Aβ42 treatment significantly reduced the average length of mitochondria and induced fragmented and punctate dot‐like shape of mitochondria. Interestingly, mitochondria in LPS‐primed microglia‐pretreated Syk inhibitor Bay 61‐3606 retained the average length and appeared as elongated morphology after Aβ42 treatment (Figure 3d,e). We also confirmed the LPS/Aβ42‐induced mitochondrial fragmentation by imaging primary microglia at different time points after Aβ42 treatment. From 3 to 24 h post‐Aβ42 treatment, we observed gradual morphological change and fragmentation of mitochondria in LPS/Aβ42‐treated microglia (Figure S2). Live imaging of LPS‐primed microglia after Aβ42 treatment further showed that Bay‐treated microglia maintain their fission/fusion balance of mitochondria after Aβ42 treatment as vehicle‐treated one, while mitochondria of LPS/Aβ42‐treated microglia get highly fragmented ([Link], [Link], [Link]). Excessive fission of mitochondria leads to mitochondrial damage and oxidative stress, including the generation of ROS, which is a well‐known activating signal for the NLRP3 inflammasome. MitoSOX staining confirmed that Aβ‐induced generation of ROS in LPS‐primed microglia was ameliorated by treatment with Syk inhibitor (Figure 3f,g). To investigate whether mitochondrial hyperfission is critical for LPS/Aβ42‐induced inflammasome activation, LPS‐primed microglia were treated for 1 h with Mdivi‐1, an inhibitor of mitochondrial division, before Aβ42 treatment. Mdivi‐1 effectively inhibited the LPS/Aβ42‐induced increases in pro‐IL‐1β, pro‐IL‐18, and cleaved caspase‐1 (Figure 3h–k).

**FIGURE 4:**
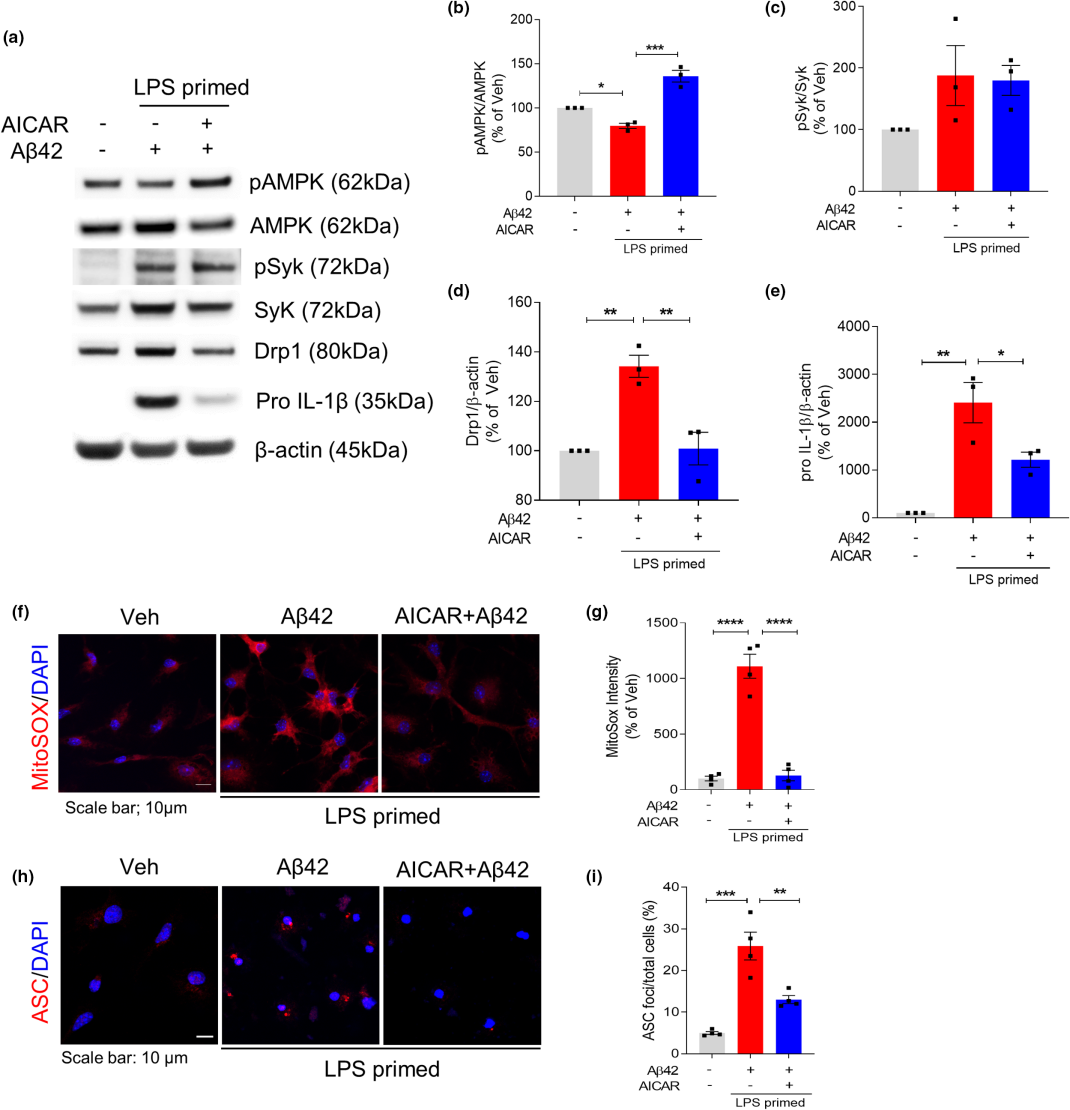
AMPK activation restores Aβ42‐induced mitochondrial hyperfission and inflammasome activation in LPS‐primed microglia. LPS‐primed microglia were pretreated with AICAR (1 mM) for 1 h and then treated with Aβ42 for 24 h. (a–e) Representative immunoblot (a) and densitometric quantifications (b–e) of the protein levels of pAMPK, AMPK, pSyk, Syk, Drp1, pro‐IL‐1β, and β‐actin in total cell lysate. (f–g) MitoSOX (red) and DAPI (blue) staining and quantification of MitoSOX fluorescence intensity. Scale bar: 10 μm. (h) Immunostaining of ASC (red) and DAPI (blue). Scale bar: 10 μm. (i) Percentages of microglia containing ASC foci were counted. Data are shown as mean ± SEM from at least three independent experiments and are analyzed by one‐way ANOVA and Tukey's multiple comparisons test. *p < 0.05, **p < 0.01, ***p < 0.001, and ****p < 0.0001

As AMPK works downstream of Syk to regulate mitochondrial function, we tested whether AMPK activator, AICAR, could restore the mitochondrial hyperfission in LPS/Aβ42‐treated microglia. AICAR rescued LPS/Aβ42‐induced AMPK inhibition but not affected Syk activation, confirming that AMPK is downstream target of Syk (Figure 4a–e). Restoration of Drp1 level (Figure 4d) and MitoSOX intensity (Figure 4f,g) by AICAR revealed that activating AMPK effectively ameliorates mitochondrial damage. Reduced mitochondrial stress by AMPK activation altered NLRP3 inflammasome activation. The level of pro‐IL‐1β (Figure 4e) and the number of ASC speck in LPS/Aβ42‐induced microglia (Figure 4h,i) were decreased by treatment of AICAR. These findings showed that Aβ42 activates Syk to inactivate AMPK and activate Akt, with this series of signaling pathways inducing mitochondrial damage and oxidative stress through mitochondrial hyperfission, which strongly activates the NLRP3 inflammasome in microglia.

### 2.4 LPS‐induced activation of the NLRP3 inflammasome is attenuated by flufenamic acid

Although NSAIDs are widely used to reduce inflammation, many aspects of their mechanism of action remain unexplained. The fenamate class of NSAIDs has been shown to selectively inhibit the NLRP3 inflammasome in immortalized mouse bone marrow‐derived macrophages (iBMDMs) (Daniels et al., 2016). To confirm that fenamate NSAIDs suppress the activation of the NLRP3 inflammasome, we first assessed key inflammasome components in primary microglia. Microglia were pretreated with acetylsalicylic acid (aspirin or ASA), ibuprofen, or FA for 1 h, followed by treatment with LPS for 24 h. Western blotting showed that pretreatment with FA, but not with ASA or ibuprofen, reduced the levels of pro‐IL‐1β, IL‐18, and caspase‐1 upon LPS stimulation (Figure S4a–d). In addition, primary microglia were incubated with various concentrations of ASA, ibuprofen, or FA, and the levels of pro‐IL‐1β were measured. Regardless of their concentration, neither ASA nor ibuprofen reduced the LPS‐induced elevation of pro‐IL‐1β (Figure S4e). In contrast, FA effectively reduced LPS‐induced expression of pro‐IL‐1β even in low concentrations (Figure S4f). In addition, real‐time PCR analysis confirmed that only FA significantly reduced the levels of IL‐1β and IL‐18 mRNAs, both of which are markedly increased by LPS. In contrast, ASA and ibuprofen had no effect on the LPS‐induced increases in IL‐1β and IL‐18 mRNA expression (Figure S4g,h). Treatment of LPS resulted in primary microglia with typically activated amoeboid‐like morphology, with these morphological changes greatly attenuated by pretreatment with FA (Figure S4i). FA alone also had no significant effect on microglial cell morphology. Furthermore, we confirmed that the anti‐inflammatory effect of FA was mediated by the Akt/AMPK pathway. Whereas treatment with ASA or ibuprofen had no effect on the phosphorylation of Akt and AMPK in LPS/Aβ42‐treated microglia, treatment with FA significantly reduced the level of pAkt and increased the level of pAMPK (Figure S4j–l). These results indicated that FA has potential anti‐inflammasome activity in microglia.

### 2.5 Flufenamic acid inhibits the NLRP3 inflammasome by activation of AMPK via inhibition of Syk

**FIGURE 5:**
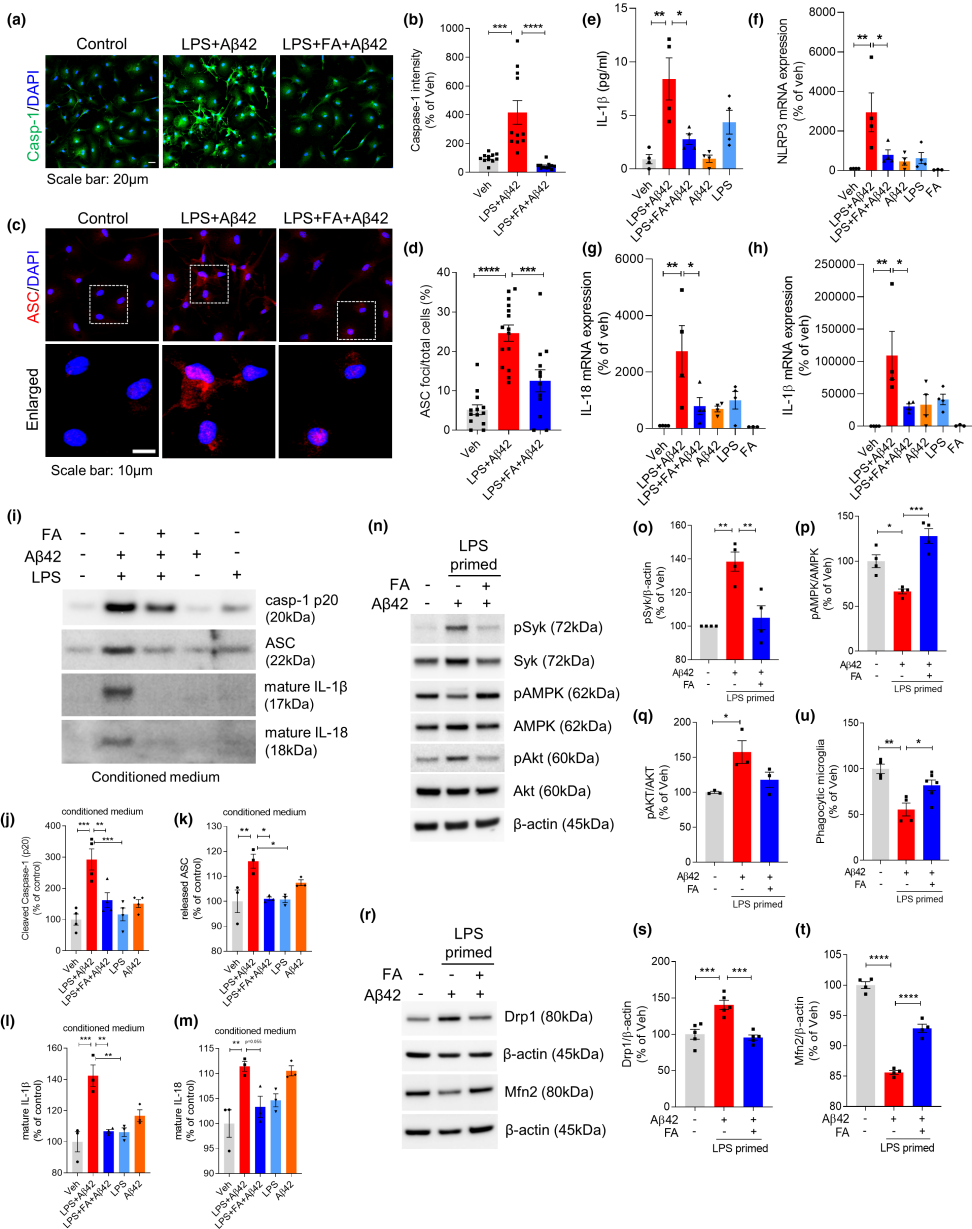
Flufenamic acid inhibits Aβ42‐induced inflammasome activation in LPS‐primed microglia. Microglia were primed with LPS (10 ng/ml) for 3 h, and stimulated with Aβ42 (4 μM) for 24 h or treated with FA (200 μM) for 1 h before Aβ42 exposure. (a) Immunocytochemical analysis of activated caspase‐1 (green) and DAPI staining (blue). Scale bar: 20 μm. (b) Quantification of the fluorescence intensity of caspase‐1. (c) Immunocytochemical analysis of ASC speck formation. Scale bar: 10 μm. (d) Percentage of cells containing ASC speck. (e) Cell culture supernatants were assayed for murine IL‐1β by ELISA. (f–h) Quantitative real‐time PCR analysis of NLRP3 (f), IL‐18 (g), and IL‐1β (h) mRNA expression. (i–m) Proteins in cultured medium were collected by TCA precipitation. Caspase‐1 (p20) (j), ASC (k), IL‐1β (l), and IL‐18 (m) were determined and quantified by immunoblotting. (n–q) Immunoblot analysis and quantification by densitometry of pSyk, total Syk, pAMPK, total AMPK, pAkt, total Akt, and β‐actin. (r–t) Mitochondrial fission‐ and fusion‐related molecules were measured by immunoblotting in LPS‐primed Aβ‐treated microglia with or without FA. (u) Relative phagocytic activity of microglia measured by bead uptake assay. Data are presented as mean ± SEM from minimum of two independent experiments, and data were analyzed by one‐way ANOVA and Tukey's multiple comparisons test. *p < 0.05, **p < 0.01, ***p < 0.001, and ****p < 0.0001

To test whether the anti‐inflammasome effect of FA is dependent on the Syk‐AMPK pathway, LPS‐primed primary microglia were treated with FA for 1 h followed by treatment with Aβ42 for 24 h. Treatment with FA dramatically attenuated caspase‐1 expression (Figure 5a,b) and ASC speck formation (Figure 5c,d). In addition, FA effectively inhibited the release of IL‐1β (Figure 5e) and reduced the levels of NLRP3, IL‐1β, and IL‐18 mRNAs compared with untreated microglia (Figure 5f–h). Protein expression of NLRP3, pro‐IL‐1β, and pro‐IL‐18 also confirmed that LPS/Aβ42‐induced NLRP3 inflammasome activation was inhibited by FA (Figure S5). Single treatment of LPS or Aβ42 was not sufficient to induce upregulation of inflammasome‐associated molecules compared with LPS/Aβ42, showing that dual signaling with LPS and Aβ is required for NLRP3 inflammasome activation in vitro. Consistent with the data from cell lysates, immunoblotting of secreted products by activated NLRP3 inflammasomes in culture supernatants of LPS/Aβ42‐treated primary microglia showed that FA significantly inhibited the secretion of cleaved caspase‐1, ASC, IL‐1β, and IL‐18 (Figure 5i–m). This inflammasome‐inhibitory effect of FA was due to its regulation of Syk activation. Specifically, FA inhibited the phosphorylation of Syk by Aβ42, thus activating AMPK and inhibiting Akt (Figure 5n–q). By regulating Syk and AMPK, FA protected against the mitochondrial fission/fusion imbalance associated with Aβ. FA pretreatment restored the expression of Drp1 and Mfn2 in LPS/Aβ42‐treated primary microglia to similar level to those in vehicle‐treated microglia (Figure 5r–t). Furthermore, the assessment of the effects of FA on phagocytic function of microglia showed that FA inhibition of inflammasome activation effectively prevented the LPS/Aβ42‐induced reduction in phagocytic activity (Figure 5u).

### 2.6 Flufenamic acid inhibits amyloid‐β and tau pathology and reverses cognitive deficits in AD mice

**FIGURE 6:**
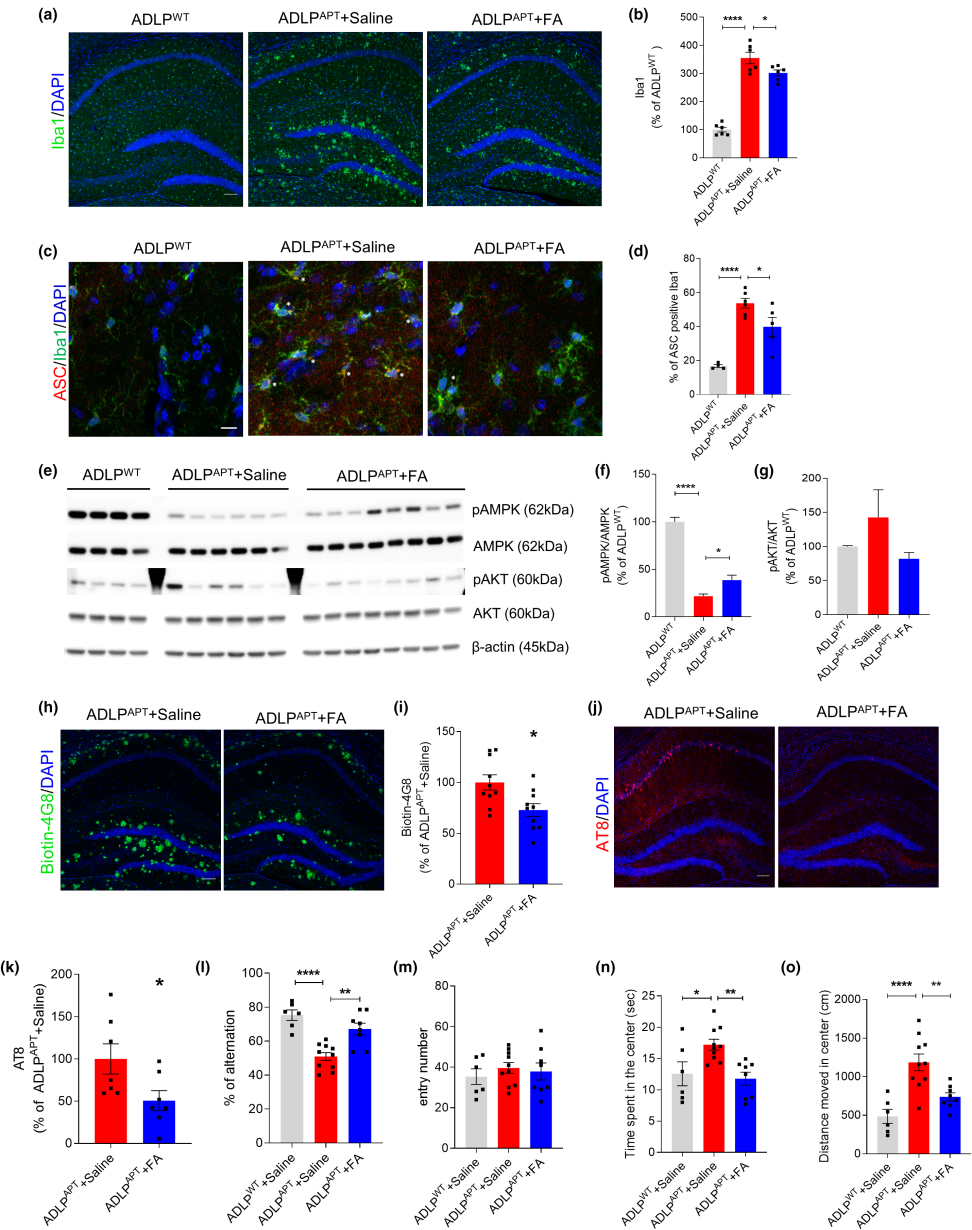
Flufenamic acid alleviates inflammation, Aβ, and tau pathology in Alzheimer's disease model. 7‐month‐old female ADLP^APT^ mice intraperitoneally injected with saline or flufenamic acid for 2 months. (a, b) Hippocampal regions of mice were immunostained with Iba1. Representative image (a) and quantification (b) of Iba1 percentage of area. *n* = 6 for each group. Scale bar: 100 μm. (c, d) ASC and Iba1 were immunostained in hippocampal region. Two images from one sectioned slice were obtained from random two regions between CA and DG of hippocampus. The number of Iba1‐positive and ASC/Iba1‐double‐positive cells was counted. A total of 80–160 Iba1‐positive cells per mouse were counted. Representative image (c) and percentage of double‐positive cells/total Iba1‐positive cells (d). *n* = 4 for ADLP^WT^+saline, *n* = 6 for ADLP^APT^+saline, and *n* = 5 for ADLP^APT^+FA. Scale bar: 10 μm. (e–g) Hippocampal homogenates of mice were used for Western blot to measure the protein levels of phospho‐AMPK (T1722448), total AMPK, phospho‐Akt (Ser723), total Akt, and β‐actin. (h, i) Amyloid plaque in hippocampus was immunostained with biotinylated 4G8 antibody (h), and its immunoreactivity was assessed by percentage of area (i). *n* = 10 for each group. Data were analyzed by the *t* test. Scale bar: 100 μm. (j, k) Hippocampus was immunostained with AT8 antibody against phosphorylated tau (Ser202/Thr205) (j). Amount of phosphorylated tau in ADLP was assessed with percentage of area (k). *n* = 7 for each group. Scale bar: 100 μm. (l, m) Memory of the mice was assessed by the Y‐maze test. *n* = 6 for ADLP^WT^+Saline, *n* = 10 for ADLP^APT^+saline, and *n* = 8 for ADLP^APT^+FA. (n, o) Open field test was used to measure the anxiety and locomotor activity of the mice. *n* = 6 for ADLP^WT^+Saline, *n* = 10 for ADLP^APT^+saline, and *n* = 8 for ADLP^APT^+FA. Data were analyzed by the *t* test for (i) and (k), and by one‐way ANOVA and Tukey's multiple comparisons test for the other data. All data were presented as mean ± SEM. *p < 0.05, **p < 0.01, and ****p < 0.0001 versus the ADLP^WT^+Saline group

Finally, we assessed whether FA could attenuate microglial inflammasome activation through the AMPK pathway in vivo, as well as the effects of FA on AD pathology. Seven‐month‐old ADLP^APT^ mice, AD model mice overexpressing both Aβ and P301L tau, were treated with daily intraperitoneal injections of saline or FA (5 mg/kg) for 2 months. In the hippocampal regions of these mice, microglial activation and the number of ASC specks were significantly greater in ADLP^APT^ than in wild‐type mice. Treatment with FA for 2 months, however, dramatically attenuated microglial activation (Figure 6a,b). In particular, FA ameliorated inflammasome activation and ASC speck formation in microglia (Figure 6c,d). Western blot analysis of hippocampal homogenates showed that the level of pAMPK was lower and the level of pAkt was higher in ADLP^APT^ than in wild‐type mice, indicating upregulated NLRP3 inflammasome of microglia in ADLP^APT^ mice. Treatment with FA restored AMPK‐Akt signaling (Figure 6e–g). In addition, FA inhibition of microglial inflammasome activation alleviated AD pathology in vivo. Immunohistochemical staining showed that the levels of amyloid plaque (Figure 6h,i) and phosphorylated tau (Figure 6j,k) were significantly lower in FA‐treated ADLP^APT^ than in control ADLP^APT^ mice. FA treatment also restored cognitive ability in ADLP^APT^ mice. In the Y‐maze test assessing cognitive function, FA treatment significantly improved memory index of ADLP^APT^ mice, reaching a similar level to control ADLP^WT^ mice (Figure 6l,m). In the open field test, FA‐treated ADLP^APT^ spent less time and moved less distance in the center zone compared with saline‐treated ADLP^APT^, indicating alleviated anxiety and locomotor activity (Figure 6n,o).

Interestingly, the effect of FA was microglia‐dependent. ADLP^APT^ mice were fed chow containing the CSF1R inhibitor PLX3397 (Spangenberg et al., 2019) or control chow for 3 months. Beginning 1 month after the start of chow treatment, the mice were intraperitoneally injected with saline or FA for 2 months. After 3 months, the number of microglia was about 70% lower in mice fed chow containing PLX3397 than in those fed control chow (Figure S6a,b). Interestingly, FA treatment did not restore AD pathology in the microglia‐depleted mice. No difference in memory was observed between PLX3397+saline‐treated and PLX3397+FA‐treated ADLP^APT^ mice (Figure S6c). In addition, immunohistochemical assays showed that FA had little effect on AD pathological features including Aβ and tau pathology (Figure S6d–g). These findings indicate that the therapeutic effects of FA in AD model mice were due to its regulation of microglial inflammasome activation.

## 3 DISCUSSION

Microglial responses to AD pathologies play primary and crucial roles in the progression of the disease. However, due to complex and heterogenetic functions of microglia, the precise mechanism by which Aβ induces microglial activation remains unclear. The present study showed that Aβ activates Syk and deactivates downstream AMPK, which mediates excessive mitochondrial fission and leads to activation of the microglial NLRP3 inflammasome, resulting in cognitive decline, amyloid plaques, and neurofibrillary tangles.

Although Aβ and tau are thought to activate the NLRP3 inflammasome by inducing lysosomal release of the inflammasome activator cathepsin (Halle et al., 2008), the upstream and downstream mediators are not clearly defined. In the present study, it suggests that Aβ activates NLRP3 inflammasome through the activation of Syk and mitochondrial impairment. Furthermore, inhibition of Aβ‐induced activation of the NLRP3 inflammasome was found to result in reductions in amyloid plaque in vivo, suggesting a positive feedback relationship between microglial inflammasome activation and Aβ pathology. Likewise, phosphorylated tau was found to act not only as an activating signal for NLRP3 inflammasome but is also affected by activated inflammasome of the microglia. The activation of the NLRP3 inflammasome has been reported to induce hyperphosphorylation of tau (Ising et al., 2019). In this study, reducing inflammasome activation attenuated the accumulation of phosphorylated tau in vivo. Because Aβ pathology is thought to affect tau pathology (Bennett et al., 2017; Blurton‐Jones & LaFerla, 2006), the reduction in tau pathology may be a secondary effect of Aβ regulation. Future studies are needed to assess the association between the NLRP3 inflammasome and tau pathology.

Syk relays signaling of immunoreceptors with the immunoreceptor tyrosine‐based activation motif (ITAM), indicating that Syk is associated with inflammatory responses of the innate immune system (Mócsai et al., 2010). Still, less is known about the role of Syk signaling in inflammasome induction, especially in microglia. Syk has been reported to activate inflammasomes in dendritic cells during anti‐fungal responses (Gross et al., 2009). The present study demonstrated that Syk mediates microglial NLRP3 inflammasome activation as a response to Aβ by regulating AMPK signaling.

Our findings also suggest a potential mechanism underlying TREM2‐mediated activation of the NLRP3 inflammasome. In microglia, TREM2 is one of the receptors with an ITAM motif that activates Syk, and Aβ has been reported to bind directly to TREM2 (Zhao et al., 2018). Therefore, TREM2 may be associated with the activation of Syk and induction of the NLRP3 inflammasome in response to Aβ. We preliminarily observed that in inflammatory condition induced by LPS, ASC speck formation was reduced in TREM2 KO mice compared with control B6 mice (data not shown). Further studies are needed to assess the involvement of TREM2 in the regulation of Syk‐AMPK‐mediated inflammasome activation. Other receptors with ITAM may also be involved in the pathway.

Aβ‐induced mitochondrial changes have been associated with a metabolic shift in microglia. Metabolic reprogramming is tightly associated with inflammatory characteristics and the function of immune cells (Ganeshan & Chawla, 2014; Jung & Mook‐Jung, 2020). Pro‐inflammatory responses of immune cells are highly dependent on their metabolic shift from oxidative phosphorylation to aerobic glycolysis, a process known as the Warburg effect (Kelly & O'Neill, 2015). Aβ has been shown to induce a metabolic shift to aerobic glycolysis in microglia (Baik et al., 2019; Galván‐Peña & O’Neill, 2014). Similarly, the present study found that LPS/Aβ42 induces the inactivation of AMPK and the activation of Akt downstream of Syk, leading to hyperfission of mitochondria. This finding is in agreement with results showing that microglial mitochondria are highly fragmented and dysfunctional in many neurodegenerative diseases (Kim & Mook‐Jung, 2019). As a well‐known regulator of metabolism, AMPK is reported to mediate mitochondrial fission/ fusion balance (Herzig & Shaw, 2018). Mitochondrial fission indicates the disruption of the electron‐transport chain and the downregulation of oxidative phosphorylation, inducing a metabolic shift to aerobic glycolysis (Katoh et al., 2017). At the same time, activation of Drp1 promotes the generation of ROS, which acts as an activating signal for the NLRP3 inflammasome (Martinon, 2010). Because metabolic reprogramming and regulation of the inflammasome are key factors of inflammatory responses in immune cells, our results suggest that activation of the Syk‐AMPK pathway is highly associated with both metabolic reprogramming and inflammasome activation, thus playing a crucial role in the pro‐inflammatory phenotype of microglia.

In this study, Syk‐induced mitochondrial fragmentation and activation of the NLRP3 inflammasome were pharmacologically regulated using a specific type of NSAID, FA. Fenamate NSAIDs, but not other classes of NSAIDs, have been reported to attenuate NLRP3 inflammasomes in BMDMs, with fenamate NSAIDs regulating NLRP3 inflammasomes by inhibiting specific chloride channels (Daniels et al., 2016). The present study found that FA effectively inhibited the Syk‐mediated pathway of inflammasome activation in microglia. Other NLRP3 inhibitors such as MCC950 and Syk‐specific inhibitors are also effective for inhibiting specific steps of Aβ‐induced inflammasome activation in microglia. At present, however, no inhibitors of microglial inflammasome have been approved for clinical use. In contrast, many fenamate NSAIDs are available for clinical use, and these inhibitors can more extensively regulate microglial metabolism and inflammatory response. The previously reported effects of FA on COX and Cl^−^ channels suggest that FA can have a synergic effect on inflammation. Importantly, FA was effective for AD pathologies only in the presence of microglia. This result not only emphasizes the importance of microglial regulation on AD pathology but also suggests that FA is less likely to have a direct effect on other cell types, reducing the risk of side effects. NSAIDs have been reported to protect against AD (Han & Mook‐Jung, 2014), but clinical attempts to apply NSAIDs as a treatment for AD have not been fully successful. Our findings suggest the need to investigate the protective or therapeutic effects of NSAIDs on human AD patients.

In summary, this study demonstrated that Syk and AMPK mediate microglial NLRP3 inflammasome activation by Aβ. Mitochondrial hyperfission is induced by activation of Syk, inducing immunometabolic reprogramming and acting as an activating signal for the inflammasome. In addition, FA may be a powerful and clinically applicable pharmacological inhibitor of inflammasome activation, ameliorating the multiple pathological features and cognitive dysfunction of AD.

## 4 EXPERIMENTAL PROCEDURES

### 4.1 Animals

5xFAD mice (Tg6799; Jackson Laboratory, stock #006554) and JNPL3 mice (TauP301L‐JNPL3; Taconic, Stock#2508 homozygote) were crossed to produce ADLP mice as described (Kim et al., 2018). At 6 months old, ADLP mice were randomly allocated into saline or FA‐treated groups. Flufenamic acid (Supelco, St. Louis, MO) or saline (DMSO added to an equal volume with flufenamic acid compound) was administered intraperitoneally, once a day, for 8 weeks. For microglia‐depleted condition, PLX 3397 was purchased from Selleckchem (Houston, USA) and formulated in chow (AIN‐76A) at 300 mg/kg. Mice were treated with PLX or control diet for 12 weeks. Intraperitoneal injection of saline or FA was started after 4 weeks of chow treatment. 6–10 mice were used for each group, and they were killed after behavioral test. Brain samples of mice were randomly allocated for staining or Western blot. The number of mice used for each experiments is written in legends of each figure. Animals were treated and maintained in accordance with the Animal Care and Use Guidelines of Seoul National University, Seoul, Korea.

### 4.2 Y‐maze test

On Day 1, after 12 weeks of chow and drug treatment, the mice were put in a Y‐maze for 3 min and were allowed to explore freely for habituation. The next day, the mice were put in the same maze for 8 min, and their arm entries were recorded. The total number of entries and the number of alternations were counted to measure the spatial memory function of the mice.

### 4.3 Open field test

Mice were placed on a white plastic board (40 × 40 cm) surrounded by white plastic walls (40 cm in height) for 30 min to freely explore while being recorded, and the video was analyzed with EthoVision XT (Noldus Information Technology, Leesburg, VA, USA). For analysis, each 40 × 40 cm unit (arena) was digitally divided into center zone (20 × 20 cm central quadrant) and peripheral zone. The distance traveled in center (cm) and time spent in center (sec) were measured.

### 4.4 Primary microglial culture

Cerebral cortices and hippocampi of postnatal day 1 ICR mouse pups were obtained by removing meninges in Hank's balanced salt solution (HBSS) from Welgene (Gyeongsan, Korea). The tissues were triturated with pipette and glass pipette in Dulbecco's modified Eagle's medium (HyClone, Massachusetts, USA) supplemented with 10% fetal bovine serum (FBS, HyClone) and 1% penicillin/streptomycin (Sigma, St. Louis, USA). Cells from a brain in 20 ml of culture medium were plated on a poly‐D‐Lysine (PDL)‐coated T‐75 flask. After 24‐h incubation at 37°C in a 5% CO_2_ incubator, the culture medium was fully changed to remove floating debris. On DIV10‐12, microglia were isolated from the mixed glial culture by tapping the flask and plated on PDL‐coated cell culture plates. The next day, the microglia were treated with LPS (10 ng/ml, L6529, Sigma) and Aβ42 (4 μM, Bachem, Bubendorf, Swiss). Bay61‐3606 (10 μM, B9685, Sigma), AICAR (1 mM, Cell Signaling, Massachusetts, USA), Mdivi‐1 (25 μM, M0199, Sigma), or FA (100 or 200 μM) was treated after LPS treatment.

### 4.5 Quantitative real‐time PCR (qPCR)

Total RNA was isolated using an RNeasy Plus Mini Kit (Qiagen, Hilde, Germany). 100 ng total RNA has been used for cDNA preparation with a Maxime RT PreMix kit (iNtRON Biotechnology, Seongnam, Korea) according to the manufacturer's protocols. Quantitative real‐time PCR was performed using SYBR Fast Mix (Applied Biosystems, Massachusetts, USA), and the following primers were used in this study: IL‐1β, IL‐18, NLRP3, and 18S ribosomal RNA (18S rRNA) was used as an internal control. The sequences of the primers were as follows: NLRP3—F‐CCC TTG GAG ACA CAG GAC TC/ R‐GAG GCT GCA GTT GTC TAA TTC; IL‐18—F‐ACT GTA CAA CCG CAG TAA TAC GG/ R‐AGT GAA CAT TAC AGA TTT ATC CC; IL‐1β—F‐TGG CAA CTG TTC CTG/ R‐GGA AGC AGC CCT TCA TCT TT; and 18S rRNA—F‐GTA ACC CGT TGA ACC CCA TT/ R‐CCA TCC AAT CGG TAG TAG CG.

### 4.6 Western Blotting

Cells were lysed in a RIPA buffer containing a cocktail of protease inhibitors and phosphatase inhibitors. 5–10 μg samples of total cell lysates were separated on NuPAGE 4–12% Bis‐Tris gels (Thermo Fisher Scientific, Massachusetts, USA) in MES buffer (Thermo Fisher Scientific) and then transferred to PVDF membranes (Merck Millipore, Massachusetts, USA). After transfer, the membranes were blocked with a 5% skim milk in TBST (Tris‐buffered saline containing 0.1% Tween‐20) for 1 h and incubated with primary antibodies at 4°C overnight. After incubation, the membranes were incubated with secondary antibodies for 1 h at room temperature. Protein bands were visualized by ECL Western Blotting Detection Reagents (AbFrontier, Seoul, Korea) and detected using a LAS‐3000 image analyzer (Fuji Photo Film, Tokyo, Japan). The primary antibodies used for immunoblotting were NLRP3 (1:1000, AG‐20B‐0014, Adipogen, San Diego, USA), IL‐1β (1:1000, AF‐401‐NA, R&D Systems, Minnesota, USA), IL‐18 (1:2000, 5180R‐100, BioVision, Milpitas, USA), caspase‐1 p20 (1:1000, AG‐20B‐0042‐C100, Adipogen), pAMPK (1:1000, 2535, Cell Signaling), AMPK (1:1000, 2532, Cell Signaling), pAkt (1:1000, 92715, Cell Signaling), Akt (1:1000, 9272, Cell Signaling), pSyk (1:1000, MA5‐14918, Thermo Fisher Scientific), Syk (1:1000, MA1‐19332, Thermo Fisher Scientific), pASC (1:1000, AP5631, ECM Biosciences, Kentucky, USA), ASC (1:1000, NBP1‐78977, Novus Biologicals, USA), Mfn2 (1:500, sc‐100560, Santa Cruz, Texas, USA), Drp1 (1:500, sc‐101270, Santa Cruz), and β‐actin (3700S, Cell Signaling). β‐Actin was used as a loading control.

### 4.7 Trichloroacetic acid (TCA) precipitation

For measurement of released protein from stimulated cells, the conditioned medium was precipitated with TCA solution. Briefly, proteins in supernatants were precipitated by adding TCA to a final concentration of 10%. Samples were vortexed and incubated on a rotator at 4°C overnight. After centrifugation at 17,900 *g* for 30 min at 4°C, supernatants were discarded and protein pellets were washed twice with ice‐cold acetone. At the end of the second wash, pellets were air‐dried and then dissolved in 2× sample buffer for Western blot. Protein samples were boiled and separated on NuPAGE 4–12% Bis‐Tris gel. Western blot analysis was performed as described above.

### 4.8 IL‐1β ELISA

Sandwich ELISA kits for mouse IL‐1β (R&D Systems, Minnesota, USA) were used for quantification of IL‐1β in cell culture supernatants according to the manufacturer's instructions.

### 4.9 Live cell imaging

Primary microglia were plated in PDL‐coated 12‐well plates. After LPS treatment for 3 h, cells were incubated with 500 nM MitoTracker (M7514, Invitrogen, Massachusetts, USA) in DMEM for 1 h to stain mitochondria. Bay or FA was co‐treated with MitoTracker. After 1 h, the microglia were treated with 4 μM Aβ42 in DMEM, and we recorded the morphological change of mitochondria during Aβ incubation with Image ExFluorer (LCI, Korea). The video was recorded for 5 h, from 2 to 7 h post‐Aβ42 treatment; snaps were taken every 3 min. The recorded video was cropped and exported using the Image ExFluorer application.

### 4.10 Immunocytochemistry

Primary microglial cells were plated on PDL‐coated coverslips. Cells grown on coverslips were washed with PBS and fixed in 4% PFA for 20 min at RT. The fixed cells were then washed with PBS and permeabilized in PBS containing 0.1% Triton X‐100 and 10% normal horse serum for 1 h. Cells were incubated with primary antibodies overnight at 4°C. After washing with PBS, the cells were incubated with Alexa Fluor 488‐ or 594‐conjugated anti‐rabbit or anti‐mouse IgG secondary antibodies for 1 h. Cell nuclei were stained with DAPI. The coverslips were imaged on a confocal laser microscope (ZEISS, Jena, Germany) with a 40× and 63× water‐immersion objective lens. The images were processed using the ZEN software. The primary antibodies used were anti‐caspase‐1 mouse antibody (1:250, AG‐20B‐0042‐C100, Adipogen), anti‐ASC rabbit antibody (1:500, NBP1‐78977, Novus), and anti‐TOM20 rabbit antibody (1:100, sc‐11415, Santa Cruz). Mitochondrial length was measured by tracing the fluorescence signals of TOM20 using the ZEN software developed by Carl Zeiss Microscopy. The average value of mitochondrial length in each group was measured and analyzed. For each group, approximately 300 mitochondria from three independent experiments were measured.

### 4.11 MitoSOX staining

MitoSOX™ Red mitochondrial superoxide indicator (M36008, Invitrogen) was dissolved in DMSO to make 5 mM stock solution. Primary microglial cells plated on PDL‐coated coverslips were incubated in HBSS with 5 μM MitoSOX™ working solution for 10 min at 37°C. After washing with PBS, cells were fixed in 4% PFA for 20 min at RT. Cell nuclei were stained with DAPI. The coverslips were imaged on a confocal laser microscope (ZEISS, Jena, Germany) with a 40× and 63× water‐immersion objective lens. The images were processed using the ZEN software.

### 4.12 Immunohistochemistry

The animals were terminally anaesthetized with a mixture of Zoletil 50 (Virbac, Carros, France) and Rompun (3:1 ratio) and perfused with PBS. The brains were removed and fixed in 4% paraformaldehyde for 48 h, followed by 72‐h incubation in 30% sucrose/PBS, and then frozen. The frozen brains were prepared in 30‐μM‐thick coronal sections for immunohistochemistry. The free‐floating sections were immersed in 70% formic acid for 20 min, permeabilized by 5% bovine serum albumin and 1% Triton X‐100 solution, and incubated overnight with primary antibodies at 4°C. Then, slices were incubated with Alexa Fluor 488‐ or 594‐conjugated IgG secondary antibodies for 1 h, followed by 10 min of DAPI counterstaining before mounting. The slices were imaged on a confocal laser microscope (ZEISS) with a 10× objective lens or 40× water‐immersion objective lens. The images were processed using the ZEN software. Images were quantified using ImageJ. The primary antibodies used were Iba1 (1:500, 234004, SYSY), ASC (1:500, NBP1‐78977, Novus), biotin‐4G8 (1:700, 800704, Biolegend), and AT8 (1:200, MN1020, Thermo Fisher Scientific).

### 4.13 Phagocytosis assay (bead uptake assay)

Phagocytic activity was measured from primary microglia that phagocytosed Fluoresbrite YG Microspheres (Polysciences, #17147). Beads were opsonized with 50% FBS in DMEM at 37°C with shaking for 30 min before the assay. After induction of NLRP3 inflammasome and drug treatment, primary microglia was treated with opsonized beads for 1 hour in dark cell incubator for the uptake of beads. All steps after the bead uptake were conducted on ice. Cells were washed twice with prewarmed 0.1 M PB and treated with prewarmed 0.25% trypsin for two minutes. 1 ml FACS buffer (PBS with 2% FBS and 0.5% sodium azide) was then added to inactivate trypsin. The cells were collected by pipetting and centrifuged at 4°C and 956 *g* for 5 min. Pellets were washed once with FACS buffer, centrifuged, and resuspended in 100 μl FACS buffer containing CD11b‐APC antibody (eBioscience, 1:1000). After incubating for 30 min at 4°C, cells were washed twice with FACS buffer and analyzed with FACSCalibur (BD Bioscience). A total of 5000 APC‐labeled cells were read, and the cells with more than two beads were gated for analysis.

### 4.14 Statistical analysis

Data are presented as mean value ± standard error of the mean (SEM). Statistical analyses performed were one‐sample *t* tests and one‐way analysis of variance (ANOVA) followed by Tukey's tests for multiple comparisons. Accepted levels of significance were *p < 0.05, **p < 0.01, ***p < 0.001, and ****p < 0.0001. Statistical analysis was performed using the software program Prism 7 (GraphPad Software, San Diego, CA, USA) for Windows.

## CONFLICT OF INTEREST

The authors declare there are no competing interests in relation to this work.

## AUTHOR CONTRIBUTIONS

IM, ESJ, and KS conceptualized the study. ESJ, KS, and JH contributed to the methodology, and visualized and investigated the study. IM supervised the study. KS and ESJ wrote the original draft and edited the manuscript. ESJ, KS, JH, WSC, HK, and HSK revised the manuscript.

## Supporting information

Figures S1–S6

Video S1

Video S2

Video S3

## Data Availability

All data that support this paper are present within the paper and/or the Supplementary Materials. The original datasets are also available from the corresponding author upon request.
