# Corrigendum: Amyloid‐β activates NLRP3 inflammasomes by affecting microglial immunometabolism through the Syk‐AMPK pathway

**DOI:** 10.1111/acel.13757

**Published:** 2022-12-19

**Jung, E. S., Suh, K., Han, J., Kim, H., Kang, H.‐S., Choi, W.‐S., & Mook‐Jung, I. (2022).** *Aging Cell*, 21, e13623. https://doi.org/10.1111/acel.13623

In Jung et al. (2022), the authors would like to correct the grant numbers listed under funding information section. The revised funding information should read as follows:

**Funding information**

This research was supported by a grant from the Korea Health Technology R&D Project through the Korea Health Industry Development Institute (KHIDI) and Korea Dementia Research Center (KDRC), funded by the Ministry of Health & Welfare and Ministry of Science and ICT, Republic of Korea (grant numbers **HU20C0187** and **HU20C0198**).

The authors would like to apologize for the inconvenience caused.

