# Supplementary material for: Amyloid‐β activates NLRP3 inflammasomes by affecting microglial immunometabolism through the Syk‐AMPK pathway
Source: Aging Cell. 2022 Apr 27;21(5):e13623. doi: 10.1111/acel.13623 (PMC9124305; doi:10.1111/acel.13623)
Supplement: Supplementary file 1 — Figures S1–S6 [file ACEL-21-e13623-s003.pdf]

Supplementary information to

Amyloid-β activates NLRP3 inflammasomes by affecting microglial immunometabolism through the Syk-AMPK pathway

Eun Sun Jung, Kyujin Suh, Jihui Han, Heyyoung Kim, Kyung-Sik Kang, Won-Seok Choi, Inhee Mook-Jung

Suppl. Figure 1. R406, syk inhibitor, protects Aβ42-induced inflammasome activation in LPS-primed microglia.

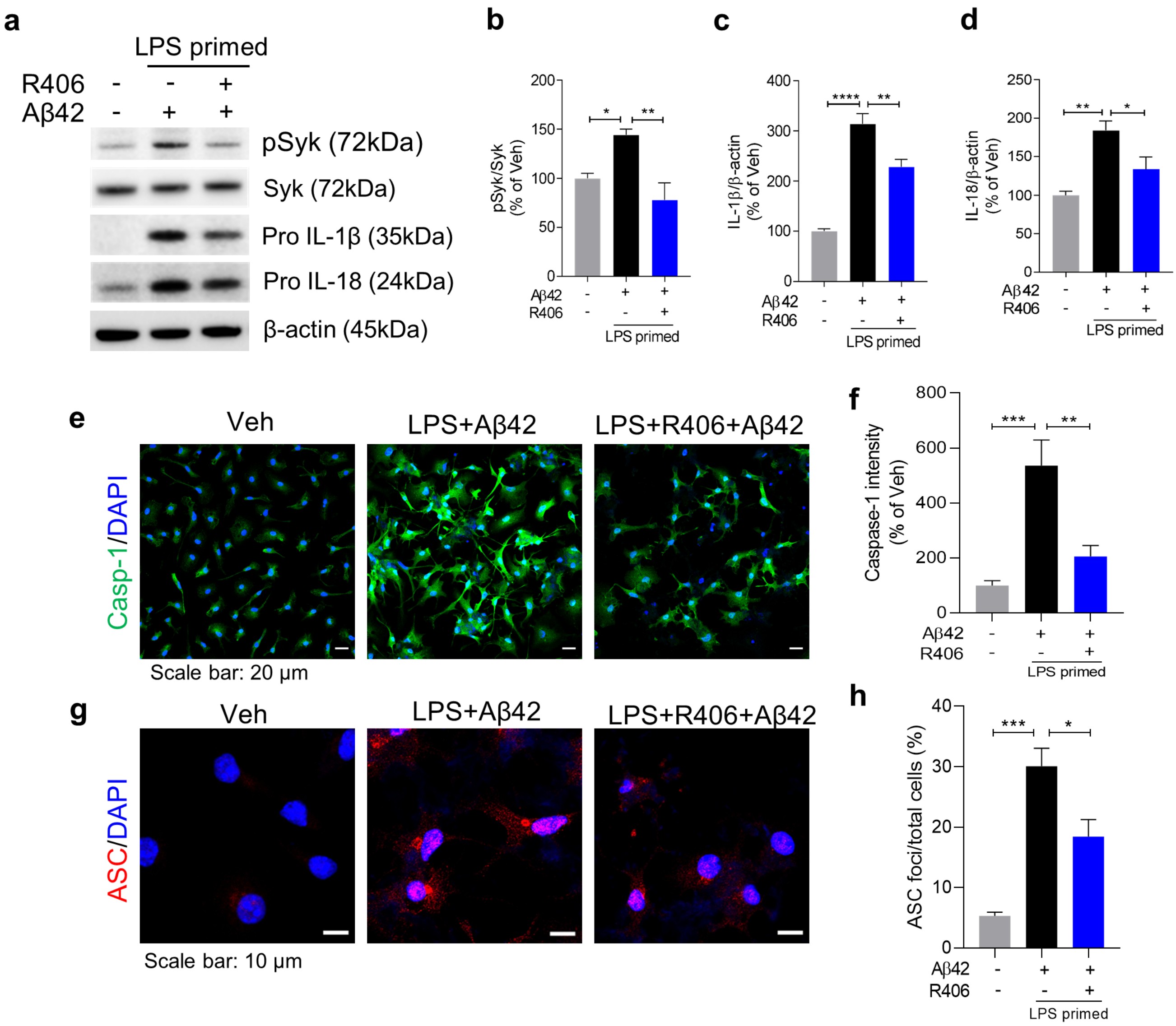

Suppl. Figure 1. R406, Syk inhibitor, protects Aβ42-induced inflammasome activation in LPS-primed microglia. LPS-primed microglia were pre-treated with Syk inhibitor R406 for 1 h followed by treatment with Aβ42 for 24 h. **(a-d)** Representative image and quantification of western blot for the pSyk, Syk, pro IL-1β, IL-18 and β-actin. **(e** and **f)** Representative immunofluorescence image of primary microglia with anti-caspase-1 (green) and quantification of immunofluorescence of caspase-1. Scale bar: 20μm. **(g** and **h)** Immunofluorescence staining for ASC (red) speck. Scale bar: 10μm. Percentages of microglia containing ASC foci was quantified. All data are represented with mean ± SEM and analyzed by one-way ANOVA Tukey's multiple comparisons test. (n=4). $^*p < 0.05$, $^{**}p < 0.01$, $^{***}p < 0.001$, and $^{****}p < 0.0001$.

Suppl. Figure 2. Bay inhibits Aβ42-induced mitochondrial fission in LPS-primed microglia.

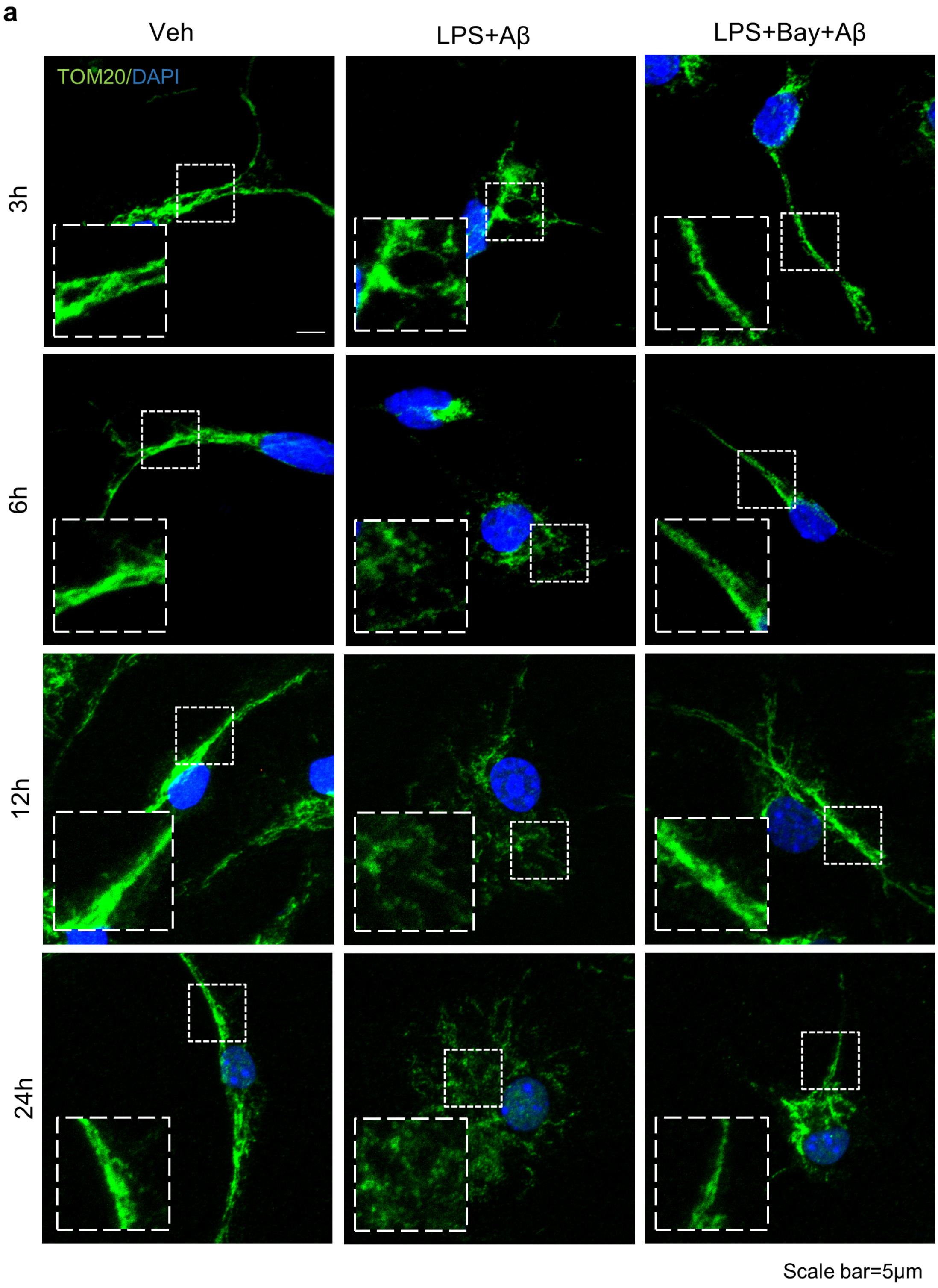

Suppl. Figure 2. Bay inhibits Aβ42-induced mitochondrial fission in LPS-primed microglia. LPS-primed microglia were pre-treated with Bay 61-3606 for 1 h followed by treatment with Aβ42 for 24 h. Cells were used for ICC at 3, 6, 12 and 24 h post Aβ42 treatment and immunostained against TOM20. (**a**) Representative images of mitochondrial morphology showing the effect of Bay 61-3606. Mitochondria and nuclei were stained with Tom20 (green) and DAPI (blue), respectively. Scale bar: 5μm.

Suppl. Figure 3. Mdivi-1 inhibits Aβ42-induced mitochondrial fission in LPS-primed micro glia.

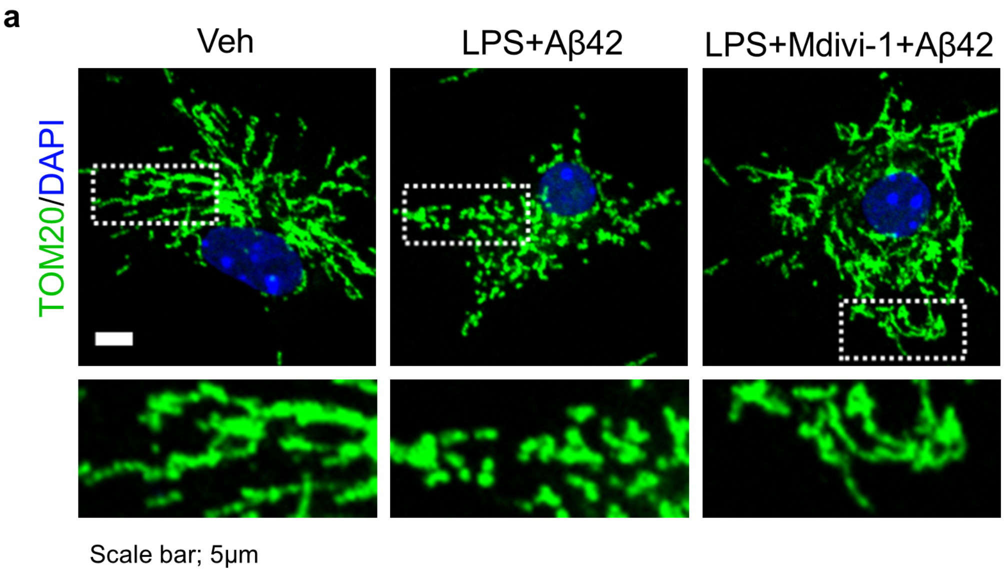

Suppl. Figure 3. Mdivi-1 inhibits Aβ42-induced mitochondrial fission in LPS-primed microglia. LPS-primed microglia were pre-treated with Mdivi-1, a selective mitochondrial division inhibitor, for 1 h followed by treatment with Aβ42 for 24 h. **(a)** Representative images of mitochondrial morphology showing the effect of Mdivi-1. Mitochondria and nuclei were stained with Tom20 (green) and DAPI (blue). Scale bar: 5μm.

Suppl. Figure 4. Flufenamic acid inhibits LPS-induced microglial inflammasome activ ation and restores altered AKT/AMPK signaling.

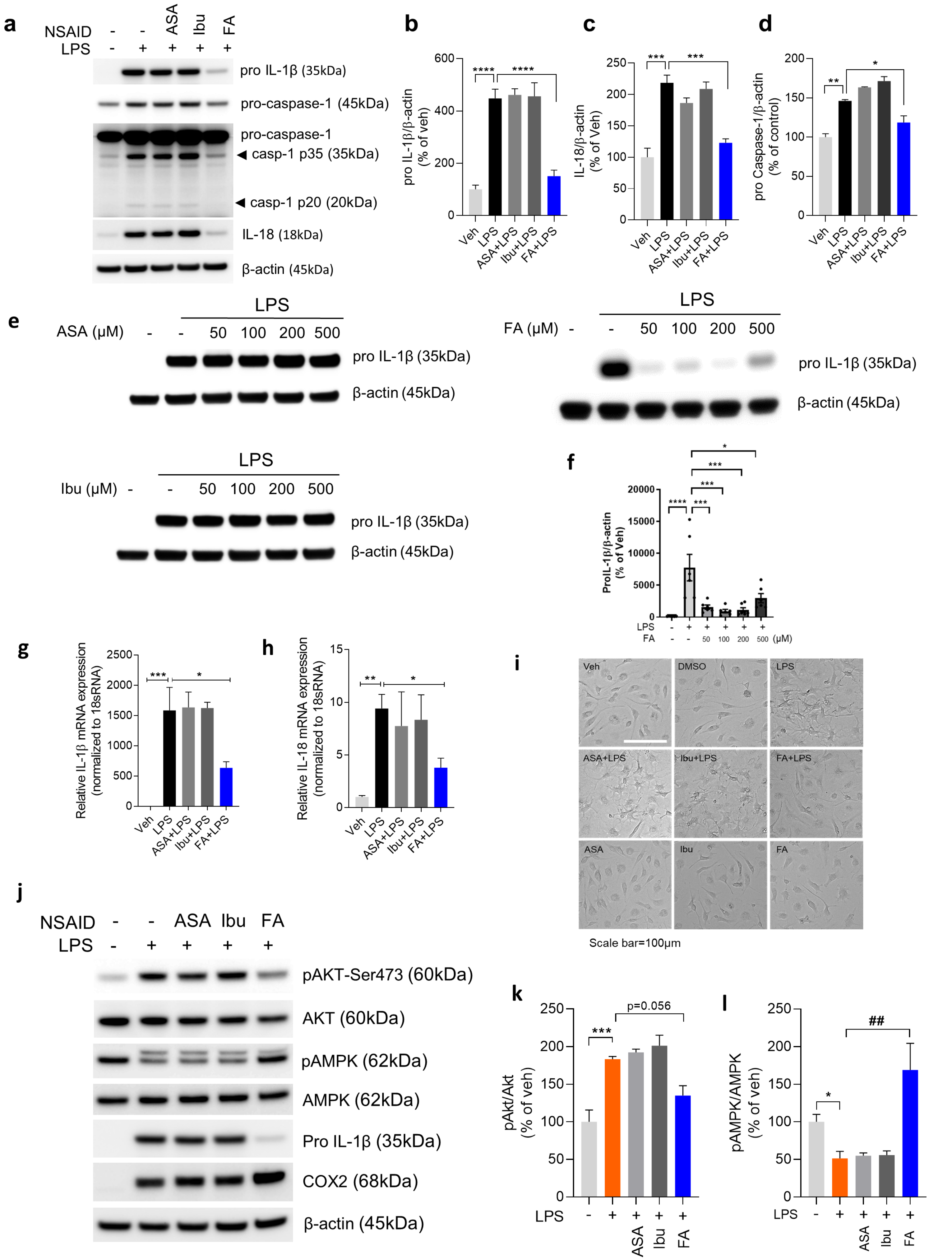

Suppl. Figure 4. Flufenamic acid inhibits Aβ42-induced microglial inflammasome activation and restores altered Akt/AMPK signaling. Primary microglia were pre-treated with acetylsalicylic acid (ASA) or ibuprofen (Ibu) or flufenamic acid (FA) for 1 h at concentration of 100 μM followed by treatment with LPS (10 ng/ml) for 24 h. **(a-d)** Whole protein lysates were analyzed for pro IL-1β, pro IL-18, Caspase-1 and β-actin. **(e)** Primary microglia were pre-treated with different concentrations of ASA, ibuprofen or FA for 1 h followed by treatment with LPS (10 ng/ml) for 24 h. The expressions of pro IL-1β protein were determined by western blot. (**f**) Quantification of western blot for FA-pretreated set. **(g** and **h)** IL-1β and IL-18 mRNA expressions were analyzed using real-time PCR. 18S rRNA was used for normalization. **(i)** Microglia morphology was examined under phase contrast microscope. **(j-l)** Primary microglia were pre-treated with NSAIDs (100 μM) for 1 hr followed by treatment with LPS (10 ng/ml) for 24 h. Protein levels of phospho-Akt (Ser723), total Akt, phospho-AMPK(T1722448), total AMPK, pro IL-1β, COX2, and actin were measured by western blot. All data are represented with mean ± SEM and analyzed by one-way ANOVA Tukey's multiple comparisons test. (n=2-4). $*p < 0.05$, $**p < 0.01$, $***p < 0.001$, and $****p < 0.0001$.

Suppl. Figure 5. Flufenamic acid attenuates Aβ42-induced inflammasome activation in LPS-primed microglia.

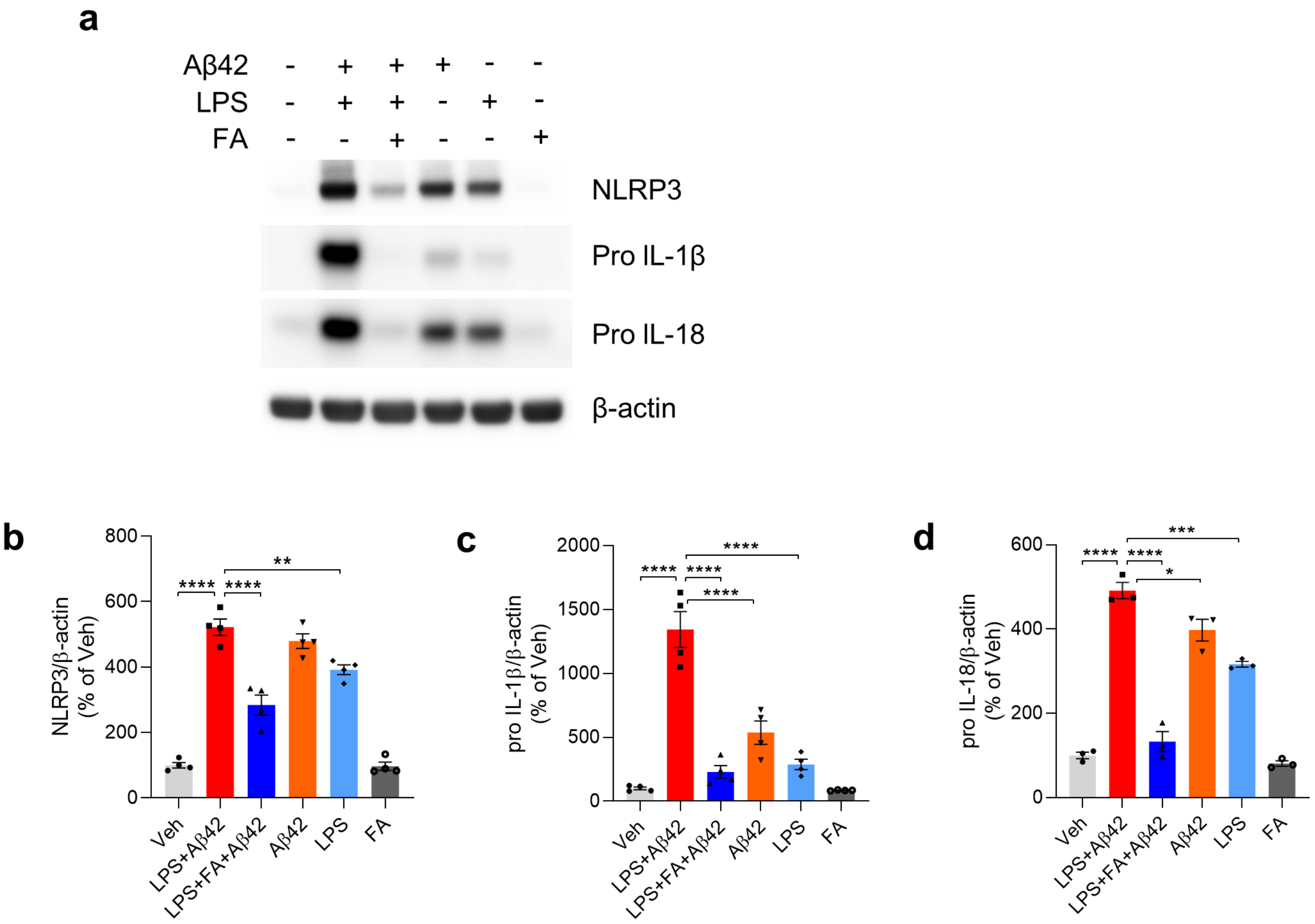

Suppl. Figure 5. Flufenamic acid attenuates Aβ42-induced inflammasome activation in LPS-primed microglia. Mouse primary microglia cells were primed with LPS (10 ng/ml) for 3 h, and washed with serum-free DMEM. LPS-primed microglia were stimulated with Aβ42 (4 μM) for 24 h or treated with FA (1μm) for 1 h before Aβ42 exposure. **(a)** Immunoblot analysis of NLRP3, pro IL-1β, pro IL-18 and β-actin in whole cells lysate. **(b-d)** Densitometric quantification of immunoblots bands. All experiments were performed at least three independent times and representative figures are shown. β-actin was used as loading control. Data are presented as mean ± SEM and data were analyzed by one-way ANOVA Tukey's multiple comparisons test. *p < 0.05, **p < 0.01, ***p < 0.001, and ****p <0.0001.

Suppl. Figure 6. Flufenamic acid alleviates AD pathology by regulating microglial activation.

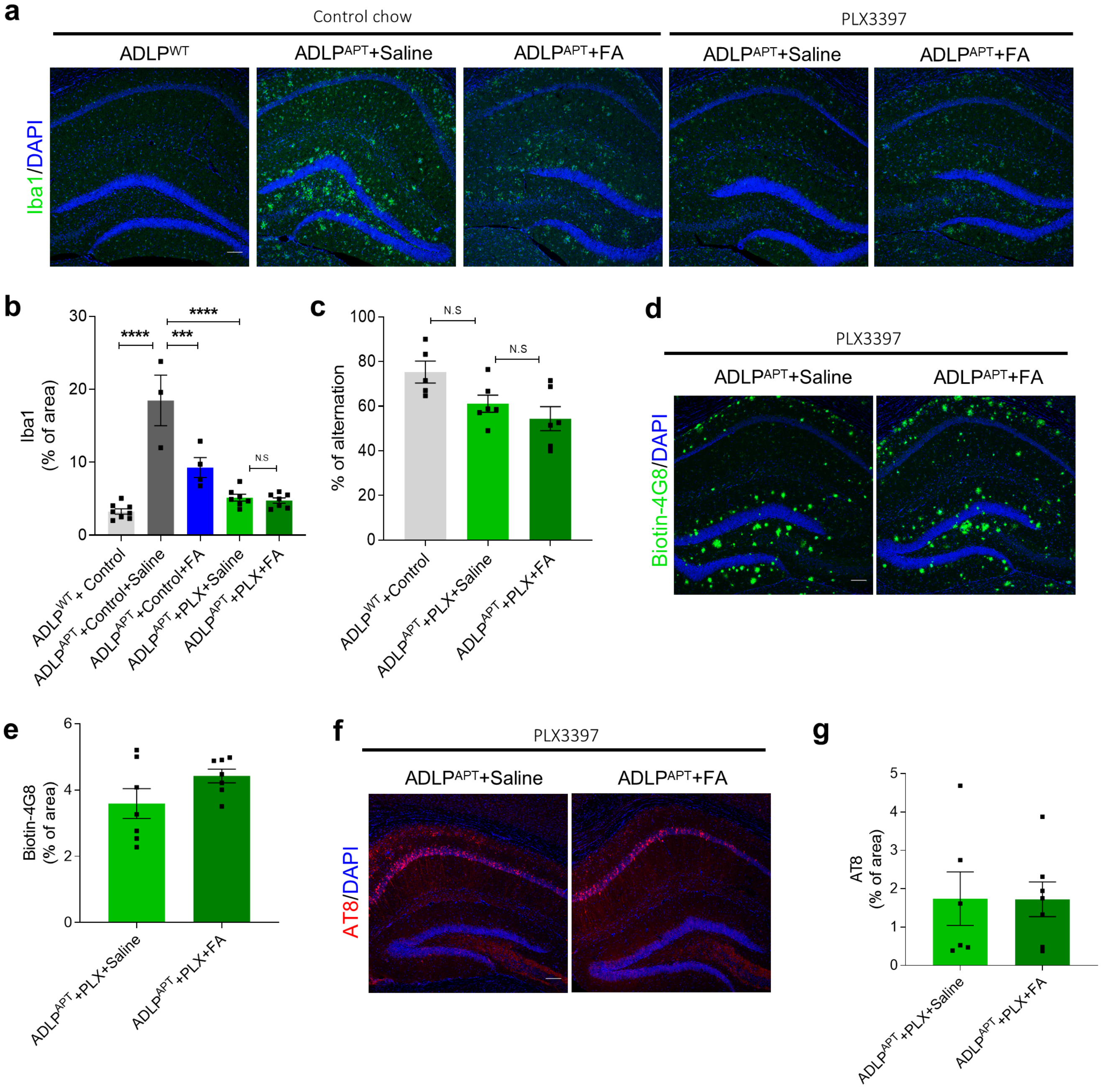

Suppl. Figure 6. Flufenamic acid alleviates AD pathology by regulating microglial activation. PLX3397 was formulated in chow (300mg/kg) and treated to 6 months-old $ADLP^{APT}$ for 3 months. Post one month from starting chow treatment, FA or saline was i.p. injected daily. (**a** and **b)** Hippocampus of mice were immunostained with Iba1. n= 8 for $ADLP^{WT}$+Saline, n=3 for $ADLP^{APT}$+saline, n=4 for $ADLP^{APT}$+FA, n= 7 for $ADLP^{APT}$+PLX+Saline and $ADLP^{APT}$+PLX+FA. % of area measured as mean ± SEM. Data were analyzed by one-way ANOVA Tukey's multiple comparisons test. Scale bar: 100μm. (**c)** Evaluation of cognitive function by Y-maze task. Alternation rates of mice were presented as mean ± SEM. n=5 for $ADLP^{WT}$+saline, n=6 for $ADLP^{APT}$+PLX+Saline and $ADLP^{APT}$+PLX+FA. (**d** and **e)** Hippocampus was immunostained with biotin-4G8 antibody against Aβ (d) and quantified by percentage of area (e). n=7 for $ADLP^{APT}$+PLX+Saline and $ADLP^{APT}$+PLX+FA. (**f** and **g)** Phosphorylated tau (Ser202/Thr205) were detected with AT8 antibody (f), and quantified by percentage of area (g). n=6 for $ADLP^{APT}$+PLX+Saline and n=7 for $ADLP^{APT}$+PLX+FA. Data were analyzed by one-way ANOVA Tukey's multiple comparisons test. *$p < 0.05$, ***$p < 0.001$ and ****$p < 0.0001$.

Suppl. Video 1. Video file showing mitochondria-labeled (green) primary microglia in vehicle condition. Scale bar, 10μm.

Suppl. Video 2. Video file showing mitochondria-labeled (green) primary microglia in LPS+Aβ condition. Asterisk indicates microglia that show strong mitochondrial fission. Scale bar, 10μm.

Suppl. Video 3. Video file showing mitochondria-labeled (green) primary microglia in LPS+Bay+Aβ condition. Scale bar, 20μm.
